# Tick–Virus–Host Interactions at the Cutaneous Interface: The Nidus of Flavivirus Transmission

**DOI:** 10.3390/v10070362

**Published:** 2018-07-07

**Authors:** Meghan E. Hermance, Saravanan Thangamani

**Affiliations:** 1 Department of Pathology, University of Texas Medical Branch (UTMB), 301 University Boulevard, Galveston, TX 77555-0609, USA; 2 Institute for Human Infections and Immunity, University of Texas Medical Branch (UTMB), Galveston, TX 77555-0609, USA; 3 Center for Tropical Diseases, University of Texas Medical Branch (UTMB), Galveston, TX 77555-0609, USA

**Keywords:** tick, flavivirus, saliva, skin, cutaneous, interface, feeding

## Abstract

Tick-borne viral diseases continue to emerge in the United States, as clearly evident from the increase in Powassan encephalitis virus, Heartland virus, and Bourbon virus infections. Tick-borne flaviviruses (TBFVs) are transmitted to the mammalian host along with the infected tick saliva during blood-feeding. Successful tick feeding is facilitated by a complex repertoire of pharmacologically active salivary proteins/factors in tick saliva. These salivary factors create an immunologically privileged micro-environment in the host’s skin that influences virus transmission and pathogenesis. In this review, we will highlight tick determinants of TBFV transmission with a special emphasis on tick–virus–host interactions at the cutaneous interface.

## 1. Introduction

**Figure 1:**
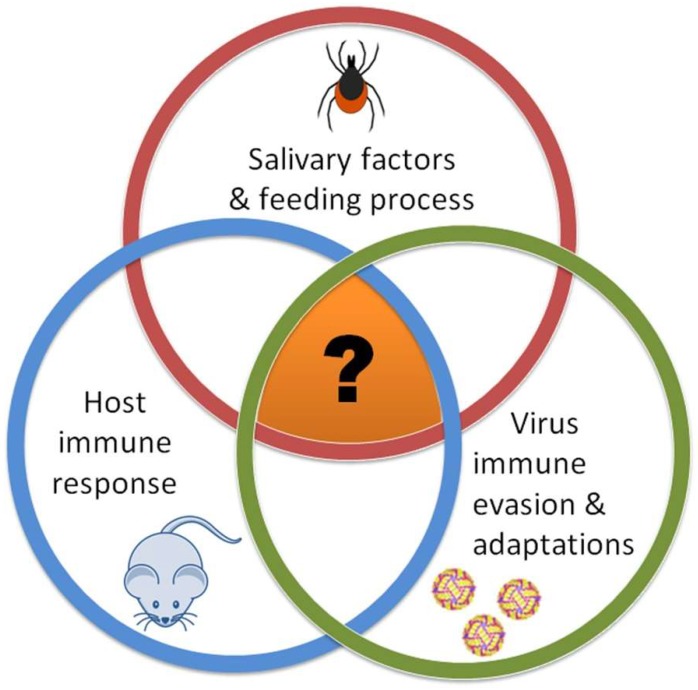
Interactions between tick-borne flaviviruses, tick vectors, and vertebrate hosts. The orange region with the question mark represents the cutaneous interface, which is the initial site where viruses gain access to a host or a vector.

The interactions between tick-borne flaviviruses (TBFVs), tick vectors, and vertebrate hosts are essential for successful tick-borne disease transmission (Figure 1). These three components interact with one another individually (tick–virus, host–virus, and tick–host) and shape the outcome of a tick-borne flaviviral infection; however, the tick feeding site is the one location where all three of these components interact together. This tripartite interaction facilitates the successful transmission and dissemination of a tick-borne flavivirus into the host. 

Skin serves as a physical barrier that provides the first line of defense against injury and infection. This complex organ possesses an array of cell populations, including immune sentinels and soluble mediators that contribute to the host’s local and systemic immune responses [1,2]. Skin is also the site where a tick initially attaches to a host and begins its lengthy feeding process. Pathogen transmission occurs during tick feeding, as skin is the first site where a pathogen gains access either to the host or to the tick vector. Therefore, the cutaneous interface is the only site in nature where TBFVs, tick vectors, and mammalian hosts contact each other simultaneously.

The redundant host defense mechanisms of the skin pose a significant threat to successful tick feeding. However, tick saliva consists of a complex array of bioactive compounds that enable the tick to remain attached and undetected by the host, to successfully blood feed, and to evade the host’s immune response [2,3,4]. Mediators of the pain and itch responses are blocked by tick salivary factors, protecting the tick from discovery and subsequent removal by the host. Tick saliva also has antihaemostatic and anti-complement activities that enable the tick to overcome host vasoconstriction, platelet aggregation, blood coagulation, and inflammation. 

Since hard ticks must remain attached to the host for extended periods of time compared to other blood-feeding arthropods, they have evolved salivary countermeasures directed against host inflammation and immune defenses. Various components of tick saliva can modulate the cutaneous innate and adaptive immune responses. Tick salivary factors are capable of altering the function of neutrophils, natural killer cells, dendritic cells, macrophages, basophils, B- and T-lymphocytes, and soluble mediators such as complement, cytokines, chemokines, and lectins [2]. As a tick feeds, salivation is not a continuous process [5], and many salivary proteins are differentially expressed during the course of feeding [3,6]. Thus, the composition of tick saliva is intricate and dynamic, enabling it to overcome the many redundancies essential to the host cutaneous defenses [7,8]. In addition to facilitating successful blood feeding, these bioactive tick salivary factors are increasingly recognized for playing a role in tick-borne pathogen transmission and establishment; therefore, there is significant scientific interest in the identification and isolation of the salivary factors responsible for these effects.

The focus of this review article will be on tick determinants of TBFV transmission in vivo. This perspective will be emphasized by highlighting the role of the cutaneous interface during the early timeline of flavivirus transmission by tick feeding.

## 2. Enhancement of Flavivirus Transmission by Tick Saliva

Saliva-assisted transmission (SAT), previously referred to as saliva-activated transmission, is the process by which bioactive salivary factors in tick saliva modulate the host environment, promoting transmission and establishment of the tick-borne pathogen. The skin feeding site of ticks is an ecologically privileged niche that can be exploited by pathogens. During SAT, tick-borne pathogens exploit the actions of tick saliva molecules at the feeding site of the tick [9]. SAT was first used to describe the enhancement of Thogoto virus (THOV) transmission by *Rhipicephalus appendiculatus* salivary gland extract (SGE) [10]. In the seminal work by Jones et al., guinea pigs were infested with uninfected *R. appendiculatus* and inoculated with a mixture of *R. appendiculatus* SGE and THOV, or with THOV alone. The number of ticks that acquired THOV from feeding on guinea pigs inoculated with virus plus SGE was approximately 10-fold greater than the number of ticks that became infected by feeding on guinea pigs inoculated with virus only, providing the first evidence that THOV transmission is enhanced by factors associated with the salivary glands of feeding ticks [10]. 

In addition to THOV, direct evidence of SAT has been demonstrated for several TBFVs [11,12]. When guinea pigs were infested with uninfected *R. appendiculatus* nymphs and inoculated with a mixture of tick-borne encephalitis virus (TBEV) plus SGE from partially fed uninfected female ticks or inoculated with TBEV alone, more guinea pigs developed a detectable viremia following inoculation with TBEV plus SGE compared to guinea pigs inoculated with virus in the absence of SGE [12]. Furthermore, the number of *R. appendiculatus* nymphs that became infected with TBEV was significantly higher in guinea pigs inoculated with TBEV plus SGE from partially fed ticks than the number of *R. appendiculatus* nymphs that became infected by feeding on guinea pigs inoculated with virus only or with virus plus SGE from unfed ticks [12]. More recently, *Ixodes scapularis* SGE was shown to enhance the transmission of Powassan virus (POWV) to naïve, immunocompetent BALB/c mice inoculated with a low dose of POWV [13]. When mice were co-inoculated with 10^3^ PFU POWV (LB strain) plus unfed *I. scapularis* SGE, the transmission and dissemination of POWV was enhanced by the presence of SGE, ultimately resulting in neuroinvasion, paralysis, and death for all mice; however, mice inoculated with 10^3^ PFU POWV in the absence of tick SGE displayed no clinical signs of infection and none succumbed to disease [13]. In these studies, the phenomenon of SAT was dependent on the inoculated virus dose, as SAT of POWV was demonstrated at the 10^3^ PFU dose of POWV but not at the 10^6^ PFU dose, suggesting that the effect of SGE on the course of disease is virus dose dependent. 

The *I. scapularis* salivary cystatin, sialostatin L2, suppresses the interferon response and enhances the replication of TBEV in mouse bone marrow-derived dendritic cells [14]. From these in vitro experiments, sialostatin L2 appears to be a novel tick salivary factor potentially responsible for SAT of TBEV; however, to date, no specific tick salivary gland factor (protein, nucleic acid, etc.) has been directly implicated in vivo for SAT of any TBFV. It is expected that a suite of salivary factors acting cooperatively are responsible for enhancing tick-borne virus transmission [9]. 

Transcriptomic and proteomic studies have demonstrated that tick genes or proteins are differentially expressed in response to pathogen infection [15]; however, the vast majority of these “omics” studies are focused on tick-borne bacterial pathogens. The effect of flavivirus infection on the salivary gland transcript expression profile was examined over a three day feeding period when *I. scapularis* nymphs were infected with Langat virus (LGTV). Differences in salivary gland transcript expression profiles were revealed between LGTV-infected and uninfected tick feeding, and the differentially regulated transcripts included Kunitz domain-containing proteins, putative secreted proteins, lipocalins, anti-microbial peptides, and transcripts of unknown function [16]. The search continues for tick salivary gland factors that promote TBFV transmission. Ultimately, the identification of such tick saliva molecules could enable the development of novel TBFV control strategies.

## 3. The Early Timeline of Flavivirus Transmission During Tick Feeding

**Figure 2:**
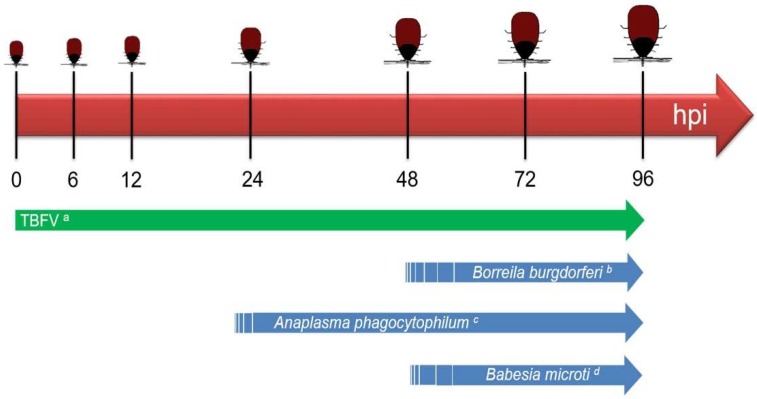
Timeline of pathogen transmission by a single infected nymphal tick. Solid region of arrow indicates experimentally validated time points of pathogen transmission. Dashed region of arrow indicates estimated timeline of earliest pathogen transmission. TBFV = Tick-borne flavivirus. ^a^ TBFVs (Powassan virus and Tick-borne encephalitis virus) can be transmitted to the host by an individual *Ixodes* tick within minutes to a few hours of tick attachment [24,25,26,27]. ^b^ The earliest documented *Borrelia burgdorferi* transmission by a single infected nymph was between 47–49 h after tick attachment [21,22]. ^c^ The earliest documented *Anaplasma phagocytophilum* transmission by a single infected nymph was observed by 24 h of tick attachment [21]. ^d^ The earliest documented *Babesia microti* transmission by a single infected nymph was observed after 54 h of tick attachment [18,28].

Hard ticks often wait many months between blood meals; therefore, the pathogens that infect hard ticks have adapted to survive these extended periods. The causative agents of Rocky Mountain spotted fever (*Rickettsia rickettsii*), Lyme disease (*Borrelia burgdorferi*), human babesiosis (*Babesia microti*), and human granulocytic ehrlichiosis (*Anaplasma phagocytophilum*) have all been shown to undergo reactivation from their dormant and essentially noninfectious state upon the next episode of tick feeding [17,18,19,20]. Transmission of *B. burgdorferi* by a single infected nymph was observed after 48 h of tick attachment, with increased transmission occurring between 72 to 96 h [21,22,23], a phenomenon that is largely attributed to the extensive reactivation phase of these spirochetes (Figure 2). 

In contrast, the timeline for transmission of TBFVs to a host appears to be much shorter than that of tick-borne bacterial pathogens (Figure 2). An *I. ricinus* tick infected with TBEV can transmit the virus from its saliva to the cement cone in the skin of a host as early as 1 h after the tick attaches and initiates feeding [24]. In an RNAseq analysis of the cutaneous TBEV-infected *I. ricinus* feeding site, TBEV reads were detected in the skin after 3 h of TBEV-infected tick feeding but not after 1 h [25]. This pattern was supported by immunohistochemical detection of TBEV antigen in the skin after 3 h of tick feeding [25]. Successful transmission of POWV (Deer tick virus, DTV-SPO) by a single *I. scapularis* nymph was shown to occur in as little as 15 min of attachment [26]. Immunofluorescence detection of POWV antigen at the skin feeding site of an individual *I. scapularis* nymph fed for three hours serves as further validation that TBFVs can be transmitted to the host within minutes to a few hours [27]. In addition to the early time points of transmission, tick-borne viruses can also be transmitted to a host over several days as a tick feeds to repletion. Experimental data suggests that in nature ticks secrete repeated “pulses” of a few infectious viral particles over the course of feeding [29]. 

Tick-borne viruses lack the complex genetic and physiologic features that enable the tick-borne bacterial and protozoal pathogens to emerge from a dormant period of metabolic inactivity to a fully infectious state [26]. The reactivation period required for some tick-borne pathogens is of public health importance because ticks infected with such pathogens provide a grace period of approximately 24 h where a minimal risk of transmission occurs if humans conduct frequent tick checks and remove an attached tick within this timeline. These differences underscore why the timeline of TBFV transmission must be considered when studying the early immunomodulatory events that occur at the skin site of flavivirus-infected tick feeding.

## 4. Early Cutaneous Immune Response to Flavivirus-Infected Tick Feeding

Skin provides the first line of defense against mechanical and environmental damage, as well as infectious agents [1,30]. It is the interface between an attached, feeding tick and a host; consequently, skin is also the first host organ that a TBFV and tick saliva encounter during the feeding process. As a tick feeds, its mouthparts and saliva come into contact with blood and lymphatic vessels, peripheral nerves, fibroblasts, keratinocytes, Langerhans cells, dendritic cells, macrophages, mast cells, natural killer cells, T lymphocytes, and soluble mediators, including cytokines, chemokines, complement, and lectins [2]. Cutaneous immune cells play a crucial role in the initial immune and inflammatory response of the host to tick feeding and pathogen transmission. 

**Figure 3:**
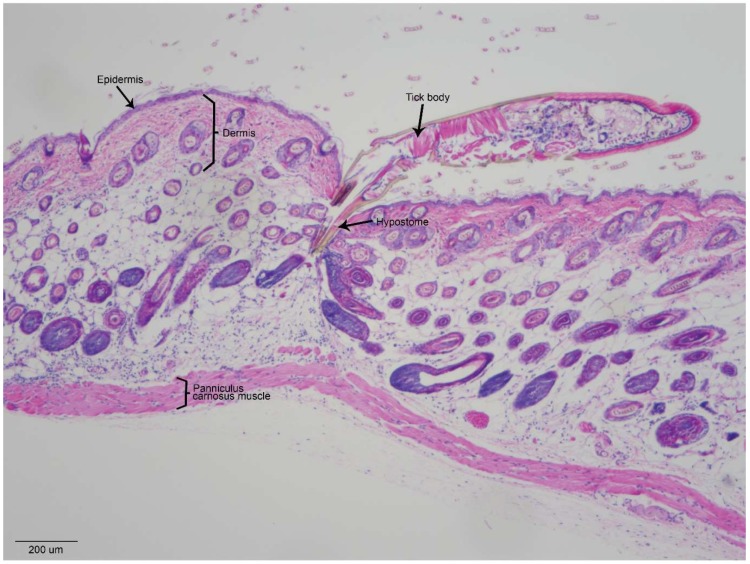
Histopathology of *Ixodes scapularis* nymphal feeding site at 3 h post infestation. The skin biopsy was harvested from the upper back of a mouse. The biopsy was fixed in 10% neutral-buffered formalin followed by decalcification prior to paraffin embedding. Five micron sections were stained with hematoxylin and eosin. Scale bar represents 200 µm.

The mouthparts of *Ixodes* species ticks, which vector the flaviviruses TBEV and POWV, are relatively long compared to other tick species (e.g., *Dermacentor* species and *Haemaphysalis* species). When TBFV-infected *Ixodes* species adults or nymphs feed on mice, the tick hypostome penetrates to the subdermal fat cells, sometimes reaching the skeletal muscle layer [25,27]. Figure 3 shows a cross-section of an *I. scapularis* nymph feeding on a naïve mouse for 3 h. The immature tick hypostome penetrates through the epidermis and dermis, with the tip of the hypostome ending amidst the subdermal fat cells (Figure 3). As a hard tick initiates feeding, its cheliceral teeth first pierce the skin and subsequently retract in a breaststroke-like motion, causing the serrated hypostome to penetrate the skin [31]. As a result of these actions, the epidermal and subdermal architecture can appear as if it is streaming toward the tick feeding site (Figure 3) [32]. Within 1 to 3 h of *Ixodes* tick attachment, inflammatory cells are recruited near the tick mouthparts, with some cell infiltrates extending into the underlying muscle (Figure 3) [25,32]. As an attached tick initiates feeding, all of these epidermal, dermal, and subdermal components, including inflammatory cell infiltrates, are in immediate contact with tick salivary molecules and flavivirus that is deposited at the tick feeding site. Because the cutaneous interface is such a complex and dynamic region during tick feeding, it is important that in vivo models (infected ticks fed on mammals) are used in experiments that seek to examine the early host immune response to flavivirus-infected tick feeding.

## 5. Cutaneous Changes at the Flavivirus–Tick–Host Interface

Since TBFVs can be transmitted to a host in less than an hour of tick feeding [24,26], the early cutaneous interactions between host immunity and initial tick-mediated immunomodulation are central to successful flavivirus transmission. From various SAT studies, it has long been suggested that tick salivary factors likely enhance virus transmission by inducing localized immunomodulation of the host, as opposed to directly affecting the virus itself [10]. A comparative gene expression analysis between POWV-infected and uninfected *I. scapularis* feeding sites was the first to use an in vivo model to characterize the host’s cutaneous immune response during the early stages of TBFV transmission [33]. *I. scapularis* nymphs, experimentally infected with POWV, were fed on mice for 3 or 6 h, and the cutaneous immune response was analyzed with pathway-specific PCR arrays. When the POWV-infected tick feeding sites were compared to the uninfected tick feeding sites, there was significant upregulation of pro-inflammatory cytokine genes (*Il1b*, *Il6*, and *Il36a*) after 3 h of tick feeding. Cutaneous gene expression analysis suggests that after 3 h of POWV-infected tick feeding, these proinflammatory cytokines contribute to the recruitment, migration, and accumulation of neutrophils and phagocytes [33]. In contrast to the 3 h time point, the majority of significantly modulated genes after 6 h of POWV-infected tick feeding were down-regulated, including several proinflammatory cytokines associated with the inflammatory response reaction, which indicates decreased recruitment of granulocytes [33]. 

**Figure 4:**
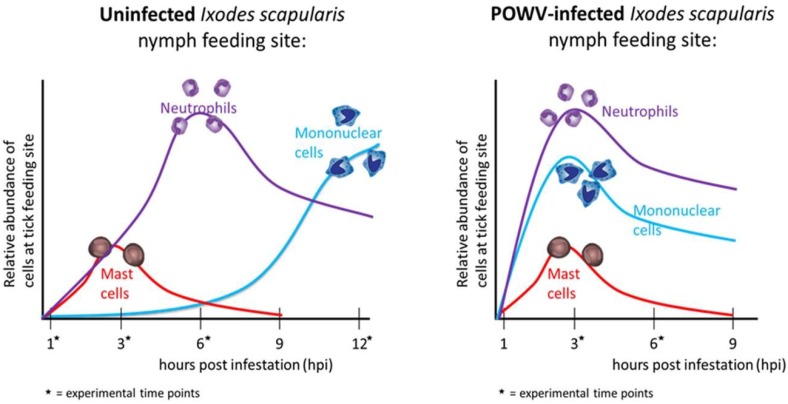
Host cutaneous immune response to tick-borne flavivirus (Powassan virus (POWV))-infected versus uninfected *Ixodes scapularis* nymph feeding. Combined results from histopathological analyses and comparative gene expression analyses demonstrate that neutrophil and mononuclear cell infiltrates are recruited earlier to the feeding site of a POWV-infected *I. scapularis* nymph versus an uninfected *I. scapularis* nymph [27,32,33]. The graphs illustrate the relative abundance of certain categories of immune cells at the tick feeding site. Asterisk represents experimental time points from these studies.

Using the same POWV–tick–host model, histopathological analyses were performed on the feeding sites of POWV-infected and uninfected *I. scapularis* fed for ≤24 h. The most distinct difference between the uninfected versus POWV-infected tick feeding sites was observed at the earliest experimental time point (3 h of tick attachment), when the infected tick feeding sites displayed higher levels of cellular infiltrates compared to the uninfected sites [27]. These cellular infiltrates consisted mostly of neutrophils and some mononuclear cells, particularly in the deep subdermal region and extending into the skeletal muscle [27]. After 6 h of tick feeding, both the uninfected and the POWV-infected sections had sparse neutrophil and mononuclear cell infiltrates, which were less than the cellular infiltrates observed in the 3 h POWV-infected sections. These histopathological findings correlate to the comparative gene expression analysis, where proinflammatory genes associated with phagocyte and neutrophil recruitment were significantly upregulated after 3 h of POWV-infected tick feeding [33]. Together, results from these studies demonstrate that neutrophil and mononuclear cell infiltrates are recruited earlier to the feeding site of a POWV-infected tick versus an uninfected tick (Figure 4) [27,33].

Using a similar in vivo model to the POWV studies, the host cutaneous immune response to TBEV-infected *I. ricinus* feeding after 1 and 3 h of tick attachment was investigated by Illumina Next Generation Sequencing and histopathology. Comparative transcriptional analysis of TBEV-infected versus uninfected tick feeding sites revealed significant upregulation of cytokines and receptors that contribute to recruitment and accumulation of immune cells, suggesting that infected ticks create an inflammatory environment at the murine cutaneous interface within 1 h of feeding [25]. Genes associated with neutrophil activation and mobilization were modulated in the presence of TBEV, indicating that an influx of neutrophils and other phagocytic inflammatory cells occurs very early at the feeding site of TBEV-infected ticks [25]. Immunohistochemistry further supported the comparative gene expression analysis of the skin lesions, demonstrating a pronounced recruitment of inflammatory cells, especially neutrophils, to the feeding site of TBEV-infected ticks compared to the uninfected tick feeding sites [25]. This in vivo TBEV study, together with the studies on the POWV-infected tick feeding sites, provide evidence of a complex, inflammatory micro-environment created in the host’s skin during the earliest stages of flavivirus-infected tick feeding [25,27,33]. The increased inflammation observed at the early feeding site of a TBFV-infected tick compared to an uninfected tick could be attributed to the TBFV itself, changes in the salivary sections in infected ticks, or a synergistic effect of both. Future experiments are needed to elucidate this phenomenon. 

## 6. The Localized Skin Site of Tick Feeding is An Important Focus for Early Flavivirus Replication and Dissemination

In natural settings, the skin is the first host organ where TBFVs gain access to either the host or to their tick vector, and infected ticks will often co-feed along with uninfected ticks on the same host [9]. Evidence of non-viremic transmission of TBEV between infected and uninfected ticks co-feeding on the same host provides insight to the mechanism of SAT and how flavivirus dissemination from the cutaneous interface occurs [11,34,35]. To mimic natural tick feeding conditions, TBEV-infected *I. ricinus* ticks and uninfected *I. ricinus* ticks were experimentally co-fed on various naïve, natural host species. The greatest numbers of TBEV-infected ticks were obtained from hosts that had very low levels of viremia (*Apodemus flavicollis* and *A. agrarius* mice) [36,37]. Findings from such studies are two-fold in importance. First, they provide compelling evidence that non-viremic co-feeding transmission of TBEV is one of the main mechanisms by which TBEV is maintained in natural foci [36,37]. Second, and perhaps most important for understanding early flavivirus infection and dissemination in the host, is these findings demonstrate that a mechanism independent of systemic viremia is responsible for flavivirus dissemination from the initial cutaneous feeding site of an infected tick. 

## 7. Cellular Targets of Flavivirus Infection at the Cutaneous Interface

To characterize TBEV-infected cells at the localized skin site of tick feeding, Labuda et al. infested laboratory strains of mice with TBEV-infected *I. ricinus* and cultured whole skin explants from the sites of tick infestation. Many leukocytes emigrated from the skin explants, and two-color immunocytochemistry revealed that TBE viral antigen was present in migrating Langerhans cells and neutrophils; furthermore, migratory monocytes and macrophages were shown to produce infectious TBEV [38]. In vitro data suggests that dendritic cell populations present at the tick feeding site are early targets of TBFV infection [39]. In a recent study, bone marrow-derived dendritic cells exposed to tick saliva enhance TBEV replication, a phenomenon that is partially attributed to the pro-survival Akt pathway [40]. Immunohistochemical analysis of TBEV-infected *I. ricinus* feeding site cross-sections demonstrated that TBEV antigen co-localizes with mononuclear phagocytes and fibroblasts, but not with neutrophils, after 3 h of infected tick feeding [25]. Immunofluorescence duplex staining of POWV-infected *I. scapularis* feeding site cross-sections revealed similar results, where POWV antigen was co-localized with macrophages and fibroblasts, suggesting that these cells are early targets of POWV infection at the tick feeding site [27]. Further research must be conducted to define what role, if any, mononuclear phagocytes and fibroblasts play in the early cutaneous establishment of TBFV infection.

Immune cells that infiltrate the skin site of tick feeding, and later migrate from such sites, can ultimately transport a flavivirus between co-feeding ticks in a process independent of systemic viremia [38]. As certain immune cells emigrate from the cutaneous tick feeding site, they are likely involved in virus dissemination. Langerhans cells are the main dendritic cell subpopulation in the epidermis. Both Langerhans cells and dermal dendritic cells serve to capture antigens in the epidermis and dermis, respectively. These cell populations mature following antigen stimulation and subsequently migrate to skin-draining lymphoid tissue, where the appropriate adaptive immune response is primed [7]. Therefore, in the experiments conducted by Labuda et al., the presence of TBE viral antigen in emigrating Langerhans cells suggests that these cells serve as vehicles for TBEV transportation to the lymphatic system, a phenomenon that contributes to overall viral dissemination. These studies illustrate the important role of localized skin infection in TBFV transmission. 

## 8. Future Directions

Many unanswered questions remain about the function of immune cells that are present at the feeding site of a TBFV-infected tick. Skin is the interface between an attached, feeding tick and a host; consequently, the cutaneous immune cells likely play a crucial role in the initial response of the host to tick feeding and virus transmission. In vivo experiments conducted at the cutaneous interface show that during the earliest stages of flavivirus-infected tick feeding, a complex, inflammatory micro-environment exists in the mammalian host’s skin, with increased recruitment, migration, and accumulation of Langerhans cells, mononuclear phagocytes, and neutrophils [25,27,33,38]. These findings indicate that TBFV-infected tick saliva immunomodulates the cutaneous micro-environment during the early stages of virus transmission to the host. In future studies it will be important to assess the function of mononuclear phagocytes, fibroblasts, and neutrophils in the early establishment and dissemination of TBFV infection. 

Systems biology is a powerful approach that can and should be utilized to examine the complex interactions between ticks, TBFVs, and vertebrate hosts (Figure 1). A major goal would be to correlate specific tick salivary molecules with defined immunological changes in the host skin, and then at the lymph nodes draining the tick feeding site. The complete assembly of the *I. scapularis* genome makes this vector species an important research model for analyses of tick–flavivirus–host interactions [41]. Recent studies of the *I. scapularis* sialome (from the Greek word sialo = saliva) have substantially advanced the identification of salivary gland components while demonstrating the very complex nature of tick saliva [3,42]. Further analysis of tick sialotranscriptomes during tick feeding would identify tick salivary molecules that modulate host immune responses and also facilitate virus transmission. Functional characterization of molecules would lead to development of anti-tick and anti-flavivirus vaccines. 

The focus of the present review was to highlight the role of the cutaneous interface during the early timeline of flavivirus transmission by tick feeding. We emphasized TBFVs in this review because they are the only family of tick-borne viruses for which in vivo studies have been conducted at the tick–virus–host interface. However, in addition to the TBFVs, there are other emerging and re-emerging tick-borne viruses distributed throughout the world. Recently, Heartland virus, Severe fever with thrombocytopenia syndrome virus, and Bourbon virus have been identified as human pathogens vectored by ticks. Knowledge obtained from *Ixodes* tick and TBFV systems cannot be extrapolated to other tick–virus systems, as each tick and pathogen is unique in modulating the host immune system. Investigations into other tick and virus systems would deepen our understanding of tick–virus–host interactions at the cutaneous interface.
